# Maternal and neonatal vitamin D status, genotype and childhood celiac disease

**DOI:** 10.1371/journal.pone.0179080

**Published:** 2017-07-07

**Authors:** Karl Mårild, German Tapia, Margareta Haugen, Sandra R. Dahl, Arieh S. Cohen, Marika Lundqvist, Benedicte A. Lie, Lars C. Stene, Ketil Størdal

**Affiliations:** 1 Norwegian Institute of Public Health, Oslo, Norway; 2 Aker Hormone Laboratory, Oslo University Hospital, Oslo, Norway; 3 Danish Center for newborn screening, Statens Serum Institut, Copenhagen, Denmark; 4 Department of Medical Genetics, University of Oslo and Oslo University Hospital, Oslo, Norway; 5 Department of Pediatrics, Østfold Hospital Trust, Grålum, Norway; Universidade de Sao Paulo, BRAZIL

## Abstract

**Background:**

Low concentration of 25-hydroxyvitamin D during pregnancy may be associated with offspring autoimmune disorders. Little is known about environmental triggers except gluten for celiac disease, a common immune-mediated disorder where seasonality of birth has been reported as a risk factor. We therefore aimed to test whether low maternal and neonatal 25-hydroxyvitamin D predicted higher risk of childhood celiac disease.

**Methods and Findings:**

In this Norwegian nationwide pregnancy cohort (n = 113,053) and nested case-control study, we analyzed 25-hydroxyvitamin D in maternal blood from mid-pregnancy, postpartum and cord plasma of 416 children who developed celiac disease and 570 randomly selected controls. Mothers and children were genotyped for established celiac disease and vitamin D metabolism variants. We used mixed linear regression models and logistic regression to study associations.

There was no significant difference in average 25-hydroxyvitamin D between cases and controls (63.1 and 62.1 nmol/l, respectively, p = 0.28), and no significant linear trend (adjusted odds ratio per 10 nM increase 1.05, 95% CI: 0.93–1.17). Results were similar when analyzing the mid-pregnancy, postpartum or cord plasma separately. Genetic variants for vitamin D deficiency were not associated with celiac disease (odds ratio per risk allele of the child, 1.00; 95% CI, 0.90 to 1.10, odds ratio per risk allele of the mother 0.94; 95% CI 0.85 to 1.04). Vitamin D intake in pregnancy or by the child in early life did not predict later celiac disease. Adjustment for established genetic risk markers for celiac disease gave similar results.

**Conclusions:**

We found no support for the hypothesis that maternal or neonatal vitamin D status is related to the risk of childhood celiac disease.

## Introduction

Celiac disease is an increasingly common autoimmune disorder, occurring in genetically predisposed individuals after ingestion of gluten from wheat and other cereals. The recent increase in celiac disease indicates that yet unidentified environmental factors may play a role in its pathogenesis.[1, 2] Several studies found that celiac disease was more common in children born during spring or summer, [3–7] suggesting that environmental factors operating with seasonal fluctuations are involved. Although a potential link to low vitamin D in the perinatal period has been proposed,[4, 5] this hypothesis has not previously been tested.

Over the last decades there has been an increased understanding that vitamin D plays an important role as a signaling component in the immune system.[8] Macrophages and monocytes express the enzymes to convert 25-hydroxyvitamin D to its active form. Vitamin D tends to suppress T-helper 1 and T-helper 17 cell responses and to facilitate T-helper 2 and regulatory T-cell responses.[8, 9]

Low vitamin D concentrations in pregnancy may adversely affect fetal development, and have been associated with long-term consequences for child health including infections.[10–13] Moreover, vitamin D deficiency in pregnancy has been associated with autoimmune diseases in the offspring, such as multiple sclerosis and type 1 diabetes.[14–16]

The main sources of vitamin D are production in the skin upon ultraviolet irradiation, as well as dietary intake from selected foods and supplements. In the liver, vitamin D is converted to 25-hydroxyvitamin D, which is the clinically relevant biomarker for vitamin D status. Vitamin D metabolites circulate mainly bound to vitamin D-binding protein (DBP). Polymorphisms in genes encoding DBP (*GC*) and enzymes involved in vitamin D metabolism are major determinants for circulating concentrations of 25-hydroxyvitamin D [17–20], while polymorphisms in the vitamin D receptor have been shown to modify the effect of vitamin D.[21, 22]

We aimed to test comprehensively the hypothesis that low concentrations of 25-hydroxyvitamin D in serial maternal and cord blood samples and genetic susceptibility markers for vitamin D deficiency are associated with increased risk of childhood celiac disease. In secondary analyses we also tested the association of season of birth and of dietary intake of vitamin D by the mother and child, with risk of celiac disease in the child.

## Materials and methods

We designed a case-control study nested within the Norwegian Mother and Child Cohort Study (MoBa). MoBa is a prospective population-based pregnancy cohort study conducted by the Norwegian Institute of Public Health recruiting pregnant women across Norway during 1999–2008, and 41% of invited women participated.[23] The current study is based on analyses of maternal samples and cord blood, data from repeated questionnaires from pregnancy through childhood and national health registries. Questionnaires are available at www.fhi.no/moba. The Norwegian Data Inspectorate and The Regional Committee for Medical Research Ethics have approved the establishment and data collection in MoBa. Written informed consent was obtained from all study participants.

### Case definition and control selection

We used the Norwegian Patient Register (NPR) and parental questionnaires completed at child age 7 or 8 years to identify children in MoBa diagnosed with celiac disease.

The NPR contains prospectively recorded nationwide data on medical diagnoses from inpatient and hospital-based outpatient care since January 1^st^, 2008. Individual patient charts or histology reports are not available in NPR. Reporting to the NPR is mandatory and linked to the governmental reimbursement system for funding of health services.

Celiac disease diagnoses in the Norwegian Patient Register for participants in the Norwegian Mother and Child Cohort Study (MoBa) were identified by register linkage using the unique personal identification number assigned to all Norwegian citizens. To reduce the risk of including false positive cases, we defined celiac disease as at least two records of the International Classification of Diseases (ICD)-10 code K90.0. This is because children during the time of celiac disease investigation may sometimes incorrectly receive a preliminary working diagnosis of celiac disease while waiting for histological or serological confirmation of the disease.

Children diagnosed with celiac disease before 2008 will be identified in the NPR if they have been followed by a specialist as recommended in the national guidelines. Additionally, in the questionnaires at child age 7 and 8 years specific questions of a diagnosis of celiac disease are answered (questionnaires available at www.moba.no).

We validated celiac disease in the MoBa cohort by contacting families with children registered with celiac disease in NPR, and excluded children with a single entry of the diagnosis in NPR (n = 185), as previously reported.[24]

**Fig 1:**
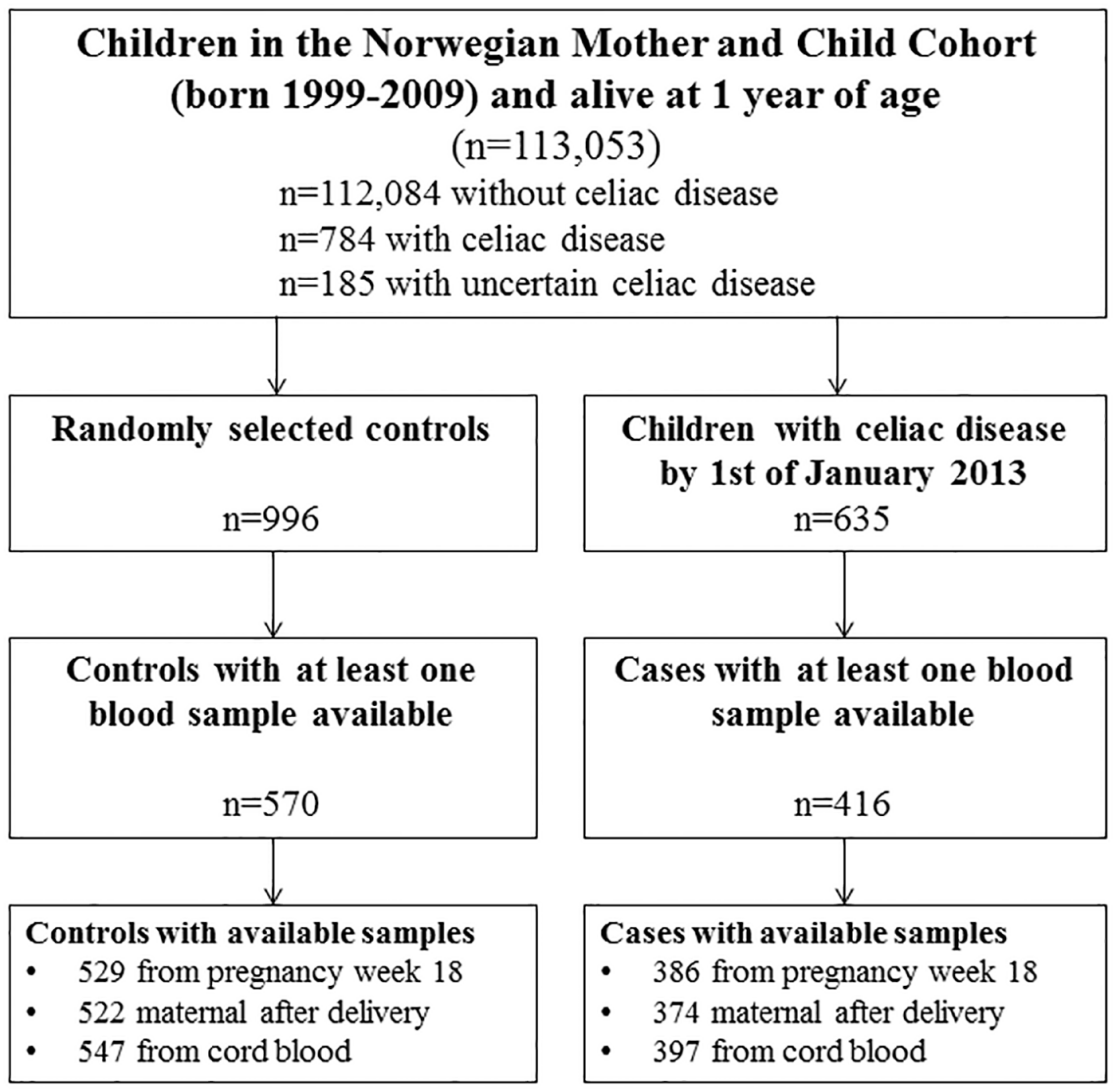
Formation of study sample for the nested case-control study. Out of 113,053 children participating in the MoBa study we randomly selected 1009 controls. Out of these, 11 had a diagnosed celiac disease and were reclassified as cases. Two were deemed to have uncertain celiac disease and excluded. In an update to cover diagnoses up to 1^st^ of January 2014, 784 had developed celiac disease (Table 1 and S1 Table).

From the MoBa cohort (n = 113,053 children), we retrieved biobanked blood samples from celiac disease cases diagnosed by 1^st^ of January 2013 (blood samples available for 416 out of 635 cases). We randomly selected 570 mother and child-pairs as controls from the whole cohort (Fig 1, flowchart). Baseline characteristics were largely similar for participants with and without available blood samples (Table 1 and S1 Table).

**Table 1:** Characteristics of participants in the nested case-control study.

	Celiac disease	
Maternal characteristics	No (n = 570) Number (%)	Yes (n = 416) Number (%)
*Age at delivery (years)*		
<25	71 (12.5)	50 (12.0)
25–34	401 (70.4)	312 (75.0)
≥35	98 (17.2)	54 (13.0)
*Parity*		
0 (first child)	247 (43.3)	169 (40.6)
1	212 (37.2)	170 (40.9)
≥2	111 (19.5)	77 (18.5)
Education level*		
≤12 years	204 (38.6)	146 (36.7)
13–15 years	213 (40.3)	168 (42.2)
≥15 years	111 (21.0)	84 (21.1)
*Smoking in pregnancy*^†^		
No	465 (87.6)	371 (92.3)
Occasionally	15 (2.8)	8 (2.0)
Daily	51 (9.6)	23 (5.7)
*Pre-pregnancy BMI (kg/m*^*2)*^*)*^‡^		
<20	58 (11.2)	51 (13.0)
20–24.9	313 (60.5)	217 (55.4)
25–29.9	108 (20.9)	83 (21.2)
> = 30	38 (7.4)	41 (10.5)
*Maternal celiac disease*		
	2 (0.4)	39 (9.4)
*Vitamin D intake in pregnancy (μg/d)* ^§^		
< 5.1	99 (25.6)	89 (29.4)
5.1–8.2	92 (23.8)	65 (21.5)
8.2–13.2	102 (26.4)	75 (24.8)
>13.2	94 (24.3)	74 (24.4)
*Norwegian origin* ^¶^		
	491 (94.2)	376 (96.7)
**Child characteristics**		
*Girls*		
	280 (49.1)	250 (60.1)
*Age at end of follow-up (mean years [SD])*		
	8.7 (2.3)	9.0 (2.0)
*Birthweight (kg)*		
<2.5	8 (1.4)	8 (1.9)
2.5–3.49	238 (41.8)	176 (42.3)
3.5–4.49	295 (51.8)	215 (51.7)
>4.5	29 (5.1)	17 (4.1)
*Preterm birth (<37 weeks)*		
	19 (3.3)	16 (3.9)
*Season of birth*		
Winter (December-February)	142 (24.9)	90 (21.6)
Spring (March-May)	154 (27.0)	115 (27.6)
Summer (June-August)	138 (24.2)	106 (25.5)
Fall (September-November)	136 (23.9)	105 (25.2)
*Number of biobank samples available*		
1	9 (1.6)	8 (1.9)
2	106 (18.6)	75 (18.0)
3	455 (79.8)	333 (80.1)

* Missing; 42 controls and 18 cases.

^†^ Missing; 39 controls and 14 cases.

^‡^ Missing; 53 controls and 24 cases.

^§^ Vitamin D intake from diet and supplements at approximately week 22 of pregnancy, expressed in quartiles defined from the MoBa cohort (S1 Table). Missing information for 183 controls and 113 cases.

^¶^ Missing; 49 controls and 27 cases.

### Additional birth registry cohort

We used an additional set of data based on all children born in Norway from 2004 to 2012 (n = 541,036 identified in the Medical Birth Registry of Norway,[25]) for assessment of season of birth in relation to risk of celiac disease. By the end of 2014, at an average of five years of follow-up, 1920 children were identified with ≥2 entries with celiac disease diagnosis in NPR.

We excluded children with a single entry of the diagnosis in NPR as explained previously (n = 597). Children participating in the MoBa study and born in 2004–2009 (n = 83 415) were included in both cohorts.

### Exposures

#### Blood sampling

Maternal blood samples were collected in EDTA tubes at hospital laboratories at enrolment around week 18 of pregnancy and again soon after delivery (0–7 days postpartum, median 1 day). Plasma was separated before shipment overnight to the MoBa Biobank. Immediately after birth, an umbilical cord blood sample was collected, shipped to the biobank and plasma separated upon arrival. All samples were stored at -80°C until analysis.[26]

#### Laboratory assays

Statens Serum Institut (Copenhagen, Denmark), a laboratory internationally certified by the Vitamin D External Quality Assessment Scheme (DEQUAS), analysed plasma 25-hydroxyvitamin D_3_ and –D_2_ using a liquid chromatography tandem mass spectrometry (LC-MS/MS, from Thermo, Waltham, MA, US). A deproteinizing solution (DPS) consisting of acetonitrile and 0.1% formic acid containing internal standard was prepared. 5 μL of serum samples, calibrators and controls were pipetted onto a 96-well μ-titer plate. 120 μL of DPS was added to precipitate proteins from the samples. The plate was sealed with a silicone plate map, shaken for 10 minutes in room temperature and centrifuged at 4000 rpm for 30 minutes at 4°C. The supernatant was transferred to a new μ-titer plate and placed under a stream of nitrogen gas until dry. 50 μl of derivatization reagent (containing PTAD) dissolved in ethyl acetate was added to the plate and shaken for 10 min before the reaction was quenched with 25 μl 99.9% ethanol. The plate was subsequently placed under a stream of nitrogen gas until dry.

The lower level of quantification (LLOQ) for 25-hydroxyvitamin D_3_ was 8.5 nmol/L, and concentrations below this were given a value of half of the LLOQ concentration (4.25 nmol/L). The inter-assay coefficient of variation for 25-hydroxyvitamin D_3_ based on repeated measurements of standards were 7.9 nmol/L. For 25-hydroxyvitamin D_2_ LLOQ was 5.8 nmol/L. Because 99.5% of the samples had concentrations below this limit, we gave the value zero for 25-hydroxyvitamin D_2_ to these samples. For the cord samples, only three samples had measurable quantities of 25-hydroxyvitamin D_2_ contributing to 16–38% of the total 25OHD. For the two maternal samples, eight and five samples had measurable 25-hydroxyvitamin D_2_ which contributed to 7–27% of total 25OHD. We used the sum of 25-hydroxyvitamin D_2_ and -D_3_ as the exposure variable, hereafter referred to as 25-hydroxyvitamin D.

The Oslo University Hospital Hormone Laboratory (Oslo, Norway) determined plasma concentration of DBP using a competitive radioimmunoassay with a polyclonal antibody (anti-Gc-globulin, Dako, Glostrup, Denmark) and purified Gc-globulin (Sigma Chemicals, St. Louis, MO, US). The measuring range (lower and upper level of quantification) was 0.7 μmol/L– 11.2 μmol/L. To obtain accurate DBP concentrations for samples outside the range of measurement, samples with concentrations > 11.2 μmol/L were further diluted, while samples < 0.7 μmol/L were less diluted. The interassay coefficient of variation was < 10% at concentrations <3.2 μmol/L and <7% at concentrations >6.8 μmol/L.

#### Genotyping assays and genetic risk scores

DNA from maternal and from cord blood was extracted and stored at the biobank. The Core Facility for Genotyping at Oslo University Hospital performed single nucleotide polymorphism (SNP) genotyping using a Custom GoldenGate assay (Illumina, San Diego, CA, US) per manufacturer’s protocol. We used 66 tagSNPs and HLA*IMP:02 to impute the main human leukocyte antigen (HLA) class II haplotypes conferring a high risk for celiac disease.[27] The imputed HLA genotype was confirmed using classical HLA genotyping with sequence-specific primers for HLA-*DRB1* (and for *DQA1* and *DQB1* in a subset of samples) at the University of Bristol.[28] The HLA risk followed the classification in high risk (two copies of the DQA1*05:01-DQB1*02:01(DQ2.5) or DQ2.5/DQA1*02:01-DQB1*02:02(DQ2.2) allele), moderate risk (DQ2.5/X or at least one of DQ2.2 or DQA1*03-DQB1*03:02 (DQ8)) or low risk (all other haplotypes) as previously described. [29] For each child we computed a non-HLA genetic risk score for celiac disease defined as the sum of risk alleles across 44 SNPs previously associated with celiac disease at genome wide significance.[30]

We genotyped four SNPs in or near four genes (rs10741657, rs6013897, rs2282679 and rs12785878 corresponding to *CYP2R1*, *CYP24A1*, *GC* and *DHCR7*) associated with 25-hydroxyvitamin D metabolism.[18–20] A vitamin D deficiency genotype score for the mother and the child was calculated by summing the risk alleles across these SNPs.[18, 19] We also genotyped two SNPs in the vitamin D receptor (rs1544410, rs11568820).[21, 22]

#### Dietary intake of vitamin D

Participants completed a food frequency questionnaire covering the period from start of pregnancy until completion around week 22 of pregnancy.[31] Total dietary intake from foods and supplements has been validated against a 4-day food diary and biomarkers.[31, 32] We divided vitamin D supplementation in the child as recorded in the 6-months- and 18-months-questionnaires into three mutually exclusive groups (daily, intermittent or no supplementation).

### Other variables

Based on previous literature we preselected variables that might influence vitamin D concentrations and risk of celiac disease as potential confounders. These included sex, age at end of study and maternal celiac disease (from questionnaire and from the Norwegian Patient Register).[33, 34] In secondary analyses we adjusted for pre-pregnant body mass index (BMI), maternal origin (indicated by whether maternal first tongue is Norwegian) and duration of full breastfeeding. [34–36]

### Preplanned sensitivity analyses

Most of the circulating vitamin D is bound to DBP, and to smaller extent to albumin. [37] In a sensitivity analysis we therefore studied the effect of the molar ratio of 25-hydroxyvitamin D:DBP, a proxy for free vitamin D. Retinoic acid, a metabolite of vitamin A, may act as an adjuvant according to an animal model of celiac disease.[38] As dietary intake and concentrations of vitamin A and D are correlated,[39] we studied whether associations were modified by dietary intake of vitamin A, as described previously.[40] Third, previous studies have suggested that risk factors for pediatric celiac disease, including season of birth, may differ by sex and age at diagnosis. [4, 5, 41] In sensitivity analyses we therefore tested whether any potential associations to vitamin D differed across sex and age-bands.

### Statistical methods

Our primary analysis focused on estimating the overall average 25-hydroxyvitamin concentration during pregnancy based on all available measurements, and its association with childhood celiac disease. To accomplish this, we first adjusted the measurements for the effects of season at sample collection,[42] and then did a two stage analysis largely following principles outlined in Chen *et al*.[43] In the first stage we used a linear mixed effects random intercept model to predict the average maternal 25-hydroxyvitamin D concentration for each woman (modelled to reflect the concentration around gestational age 25, the average gestational age at collection of all samples). In the second stage we used this average as the predictor in a logistic regression analysis with celiac disease in the child as the outcome, and finally computed 95% confidence intervals (CI) accounting for the total variance at both stages using bootstrapping with 2000 repetitions. Our primary analysis used 25-hydroxvitamin D as a continuous variable to test for linear trend, but we secondarily tested the linearity assumption by plotting results from categorical analysis and testing quadratic terms. We calculated prior to the study that 400 cases and 600 controls would provide 98% statistical power to detect a linear trend across all values if the true odds ratio for the upper versus lower quartile was 2.0. We used Stata (V14, StataCorp., College Station, TX, US) for the statistical analyses.

## Results

**Fig 2:**
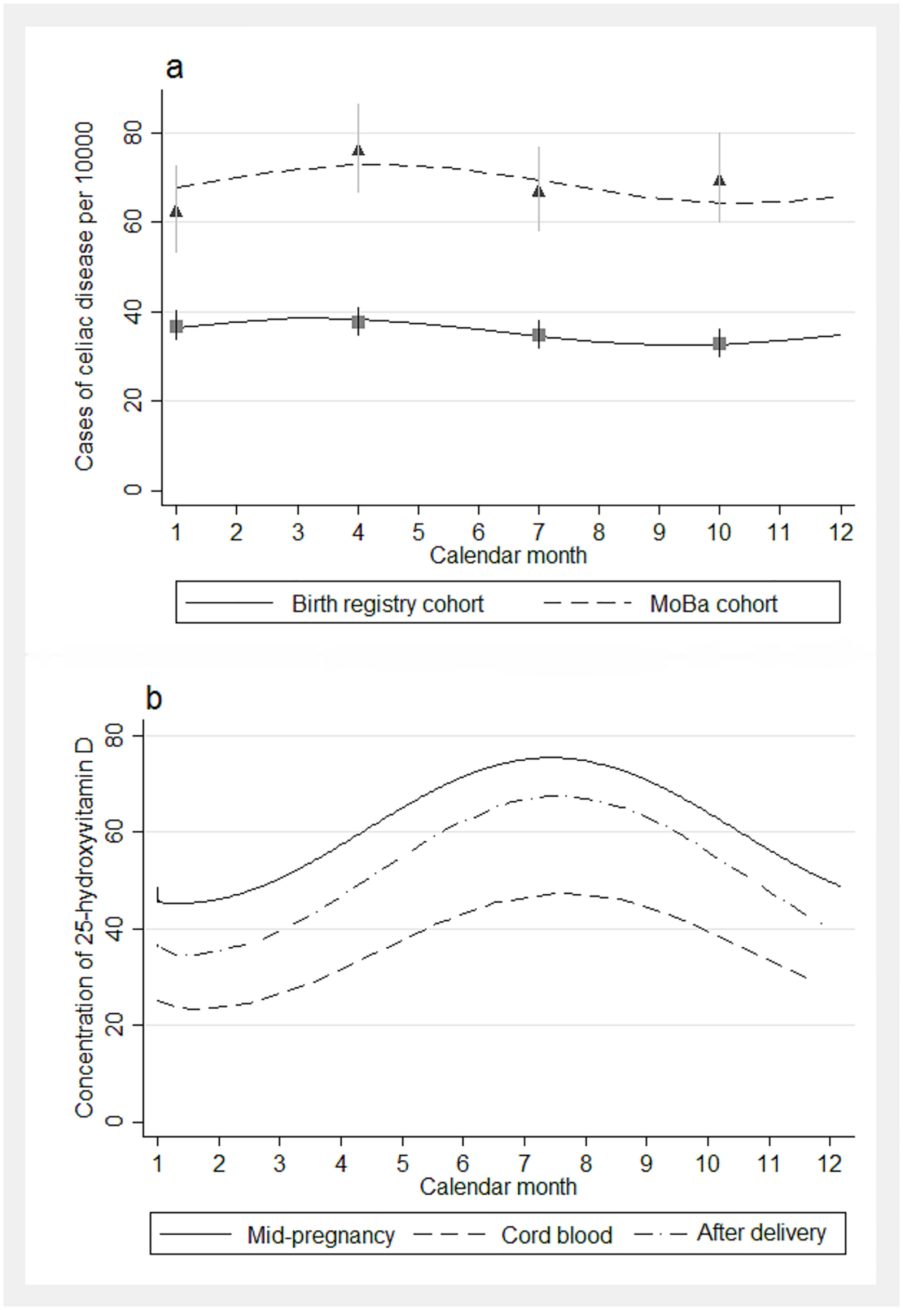
Risk of celiac disease by season of birth and seasonal variation of vitamin D status. a). Cumulative incidence of celiac disease by season of birth in the MoBa cohort (mean age 9.0) and in a birth registry cohort (mean age 5.5 years). b). Average concentration of 25-hydroxyvitamin D nmol/L by calendar month of year at time of sample collection.

Children in the MoBa cohort born during spring had a slightly increased risk for celiac disease compared to those born during winter (odds ratio 1.22, 95% CI 1.00 to 1.49, S1 Table). In the register-based cohort, season of birth was similarly associated with the risk of celiac disease at borderline significance (p = 0.05, Fig 2a).

In MoBa cohort odds ratio 1.22 (1.00–1.49) between season with highest (spring; March-May) vs lowest incidence (winter; December-February).

In birth registry cohort odds ratio 1.14 (1.01–1.30) between season with highest (spring; March-May) vs lowest incidence (fall; September-November).

Smooth lines are predictions from cosinor regression models. Squares and triangles are point estimates for 3-month categorization of season of birth, and vertical lines are 95% confidence estimates.

### 25-hydroxyvitamin D and risk of childhood celiac disease

The concentrations of 25-hydroxyvitamin D peaked during fall, with a nadir during spring months (Fig 2b). Mean maternal mid-pregnancy 25-hydroxyvitamin D among controls was 59.7 nmol/L, compared to 50.1 nmol/L after delivery; mean concentration in cord plasma was 35.4 nmol/L and strongly correlated with maternal postpartum samples (r = 0.77).

**Fig 3:**
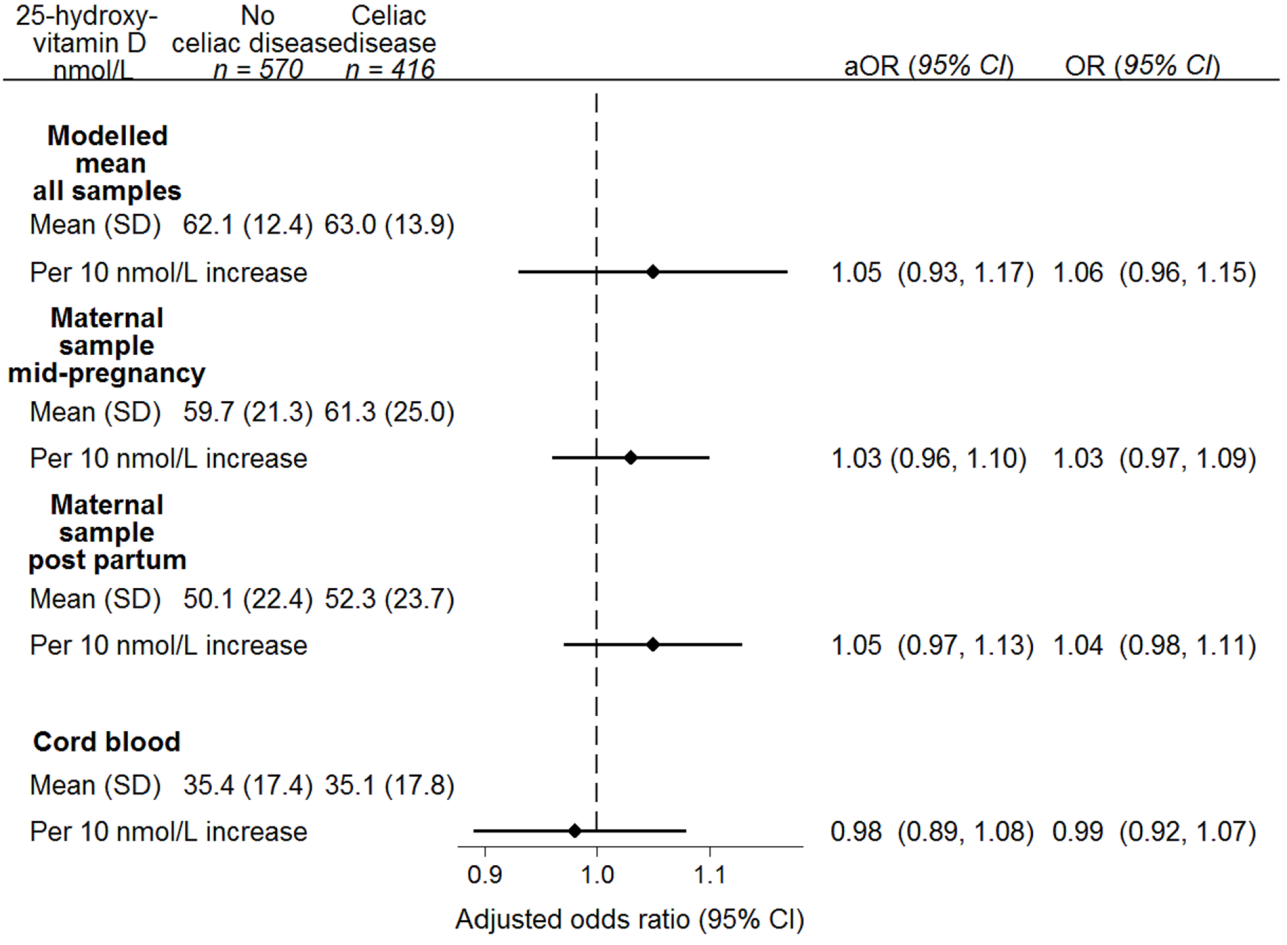
Association between maternal and cord blood 25-hydroxyvitamin D* and risk for offspring celiac disease. * Corrected for seasonal variation at month of sampling. aOR; adjusted odds ratio, OR; odds ratio, CI; confidence interval. The primary analysis used the modelled mean concentration of 25-hydroxyvitamin D across all available samples, predicted from mixed effects random intercept model. P for trend = 0.26. Maternal samples from week 18: cases, n = 386; controls n = 529. P for trend 0.78 Maternal samples after delivery: cases, n = 374; controls, n = 522. P for trend 0.50. Samples from cord blood: cases, n = 397, controls, n = 547. P for trend 0.60. Odds ratios adjusted for sex, attained age, maternal celiac disease, non-HLA genetic risk score and HLA haplotype.

The overall average maternal 25-hydroxyvitamin D in cases was 63.0 nmol/L compared to 62.1 nmol/L in controls. The unadjusted odds ratio per 10 nmol/L increase was 1.06 (95% CI: 0.96–1.15, Fig 3). We also found no associations of 25-hydroxyvitamin D concentrations separately in the samples taken at mid-pregnancy, postpartum and cord blood with risk of celiac disease in the children (Fig 3). Adjustment for HLA and non-HLA conferred celiac disease risk did not appreciably change the results (Fig 3). Further assessment for risk of celiac disease across the range of concentrations of 25-hydroxyvitamin D did not reveal any non-linear associations (S1 Fig).

### Dietary vitamin D and celiac disease

Vitamin D intake in pregnancy was not associated with the risk of celiac disease in offspring, neither in the case-control sample nor in the entire MoBa cohort (S1 Table). We found no association for use of vitamin D supplementation during the first 18 months of life with later celiac disease. Adjustment for child vitamin D deficiency genotype score and for genetic risk for celiac disease yielded only marginally changed estimates (Table 2).

**Table 2:** Associations of vitamin D deficiency genotype score in mother and child, use of postnatal vitamin D containing supplements, and risk of childhood celiac disease.

	**Celiac disease**		**Unadjusted odds ratio for celiac disease**	**Adjusted odds ratio for celiac disease** ^‡^
	**No (n = 559)**	**Yes (n = 402)**	**(95% CI)** ^†^	**(95% CI)**
***Maternal vitamin D deficiency genotype score*,** *				
***n (%)***				
**<2 risk alleles**	79 (14)	101 (25)	1.00 (ref.)	1.00 (ref.)
**2 risk alleles**	140 (25)	114 (28)	0.88 (0.58, 1.32)	0.88 (0.48, 1.63)
**3 risk alleles**	173 (31)	72 (18)	0.63 (0.42, 0.94)	0.88 (0.48, 1.61)
**≥ 4 risk alleles**	167 (30)	115 (27)	0.75 (0.50, 1.12)	0.92 (0.50, 1.67)
***Child’s vitamin D deficiency genotype score*,** *	No (n = 566)	Yes (n = 395)	Unadjusted odds ratio for celiac disease	Adjusted odds ratio for celiac disease^†^
***n (%)***			(95% CI) ^§^	(95% CI) **
**<2 risk alleles**	89 (16)	51 (13)	1.00 (ref.)	1.00 (ref.)
**2 risk alleles**	140 (25)	122 (31)	1.50 (0.98, 2.28)	1.68 (0.90, 3.10)
**3 risk alleles**	172 (30)	109 (28)	1.11 (0.73, 1.69)	1.07 (0.58, 1.98)
**≥ 4 risk alleles**	165 (29)	113 (29)	1.23 (0.81, 1.88)	1.26 (0.68, 2.32)
***Child’s vitamin D supplementation***	n (%)	n (%)	Unadjusted odds ratio for celiac disease	Adjusted odds ratio for celiac disease^||^**
			(95% CI)	(95% CI)
**No use**	68 (17)	52 (17)	1.00 (ref.)	1.00 (ref.)
**Occasional use**^§^	235 (60)	191 (62)	1.06 (0.71, 1.60)	0.94 (0.55, 1.61)
**Daily use**	91 (23)	65 (21)	0.93 (0.58, 1.51)	0.98 (0.53, 1.81)

* Four common gene variants associated with vitamin D deficiency categorized into quartiles according to number of risk alleles among controls. 1^st^ quartile represents <2 risk alleles, 2^nd^ quartile 2, 3^rd^ quartile 3 and 4^th^ quartile ≥4 risk alleles for vitamin D deficiency. Missing genotype for 4 controls and 21 cases among the children, and missing for 11 controls and 14 case mothers.

^†^ Crude OR for offspring celiac disease per maternal risk allele for vitamin D deficiency 0.94 (95% CI 0.85 to 1.04), p-value for trend 0.23.

^‡^ Adjusted for child sex, attained age by 2013, maternal CD, use of vitamin D supplements, non-HLA genetic risk score and HLA haplotype.

^§^ Crude OR for celiac disease per child risk allele for vitamin D deficiency 1.00 (95% CI 0.90 to 1.10), p-value for trend 0.95.

^¶^ Use of supplement containing vitamin D either from 0–6 months or from 6–18 months, or non-daily use in either period. Missing information for 176 controls and 108 cases.

^||^Adjusted for child sex, attained age by 2013, maternal CD, child vitamin D genotype, HLA haplotype and for non-HLA genetic risk score.

**OR per category increase 0.99 (95%CI 0.73 to 1.35), p-value for trend 0.96

### Vitamin D deficiency genotype score and celiac disease

Maternal and child vitamin D deficiency genotype score did not predict the risk of celiac disease in the offspring, Table 2. Neither were genetic variants at the vitamin D receptor gene associated with later celiac disease (S2 Table).

### Secondary and sub-group analyses

We repeated our primary analysis using the molar ratio of 25-hydroxyvitamin D:DBP,[37] and again found no association (adjusted OR 1.02, 95% CI 0.99 to 1.04 per unit increase, S2 Fig). Repeating the primary analysis without adjusting for season at blood sample collection or restriction to only parental-confirmed celiac cases did not change any of the conclusions (S3 Table).

Adjusting our primary analysis for duration of full breastfeeding, pre-pregnancy BMI and maternal origin only marginally changed the main estimates (S3 Table). While self-reported intake of vitamin A in pregnancy correlated with vitamin D intake (r = 0.49, p<0.001), it was not associated with offspring celiac disease (p for trend = 0.82). Adjustments for vitamin A changed the association for 25-hydroxyvitamin D and celiac disease marginally, and with no signs of effect modification (p for interaction 0.27, S3 Table).

There were no associations between 25-hydroxyvitamin D in pregnancy and celiac disease in subgroups defined by sex, age at celiac disease diagnosis and presence of HLA-DQ2.5 (S3 Table).

## Discussion

Our comprehensive analysis provided no support for the hypothesis that gestational or early life vitamin D status plays a role in the development of childhood celiac disease. The consistency of our observations and the robustness to adjustments for potential confounders including genetic risk for celiac disease, strongly argues against vitamin D as a risk factor for celiac disease.

Although a potential role of vitamin D has been hypothesized by others,[4, 5] this study is to our knowledge the first of its kind to test this hypothesis. Among the strengths of the study were the repeated measurements of 25-hydroxyvitamin D and inclusion of genetic risk factors for both vitamin D deficiency and celiac disease. This study also included prospectively collected information on vitamin D intake and several potential confounders.

The study also had some weaknesses. We did not have blood samples from the children after birth to study 25-hydroxyvitamin D, but on the other hand, we had information on the child’s vitamin D deficiency genotype score and prospectively collected information on vitamin D supplements in the first 18 months. This provided no support of the hypothesis that the child’s genetic risk for vitamin D insufficiency or intake was associated with celiac disease. An important strength of the vitamin D deficiency genotype score is that genetic factors are not confounded by behavioral and other biases.[44] The wide uncertainty ranges for the associations with vitamin D deficiency genotype score and celiac disease also calls for caution in the interpretation due to our limited sample size. We cannot rule out that the proportion of refusals in the initial cohort and the proportion without available blood samples could have resulted in some selection bias. Thus, a weak association cannot be completely ruled out from a single study like ours with a potential for bias. Furthermore, we did not have available information on sunlight exposure. In addition to direct effects on 25OHD, sunlight exposure could potentially act through other pathways.

If vitamin D were associated with later celiac disease, we would assume these observations more likely to be present in the perinatal period compared to later in life due to the observed associations to season of birth. The increased risk for celiac disease with spring and summer birth in various cohorts, albeit inconsistent,[3–7] was small in our two cohorts. Some of these previous studies have observed this association only in subsets of the studied population, calling for cautious interpretation.[33] While our study suggests that vitamin D status early in life is unlikely to account for the observed association between season of birth and risk of celiac disease, other factors with a seasonal variation cannot be ruled out. For example, we have previously found that MoBa-children with a high infection frequency in early life have an increased risk for later celiac disease. Speculatively, the potential seasonal effects may instead operate through infections occurring during windows of increased vulnerability.[24] Our cohort is still young, and vitamin D may play a role for celiac disease diagnosed later in life. However, our sensitivity analyses did not detect age as an effect modifier.

We regard the risk of misclassification of diagnosed celiac disease as low, according to our previous validation of celiac disease in the cohort. Restricting our analyses to confirmed cases resulted in essentially unchanged estimates. Screening for celiac disease could not be done in our study. However, assuming a prevalence of 1% of undiagnosed disease, it is unlikely that false-negative disease status could have influenced our results more than marginally.[45]

In some ethnic groups vitamin D deficiency is highly prevalent, with lower average 25-hydroxyvitamin D concentrations than in our sample.[46] Our findings are generalizable for populations similar to ours, where the intake of vitamin D is lower than recommended.[47]

A potential preventive effect of vitamin D in utero or in early life would have immediate clinical and public health implications, but a final test of this hypothesis would imply a large, well conducted randomized controlled trial with vitamin D supplementation to pregnant mothers. Although we cannot formally exclude the existence of unmeasured confounders, our study does not encourage the initiation of such a trial.

In conclusion, in this comprehensive study 25-hydroxyvitamin D in pregnancy, genetic susceptibility for vitamin D deficiency and early postnatal vitamin D supplementation were not associated with pediatric celiac disease. These consistent results argue against a clinically meaningful role of perinatal 25-hydroxyvitamin D in childhood celiac disease.

## Supporting information

S1 Fig Risk for offspring celiac disease by centiles 25-hydroxyvitamin D modeled for gestational week 25 (A) and for individual sample points (B-D). Density plot for 25-hydroxyvitamin D concentrations among cases (solid line) and controls (dashed line) modeled for gestational week 25 (E) and for individual sample points (F-H).
(DOCX)

S2 Fig Odds ratios for offspring celiac disease according to ratio of 25-hydroxyvitamin D:D-vitamin binding protein for week 25 and for individual time points. DBP, D-vitamin binding protein; aOR, adjusted odds ratio; OR, odds ratio; 95% CI, 95% confidence interval. The primary analysis used the modelled mean ratio of 25-hydroxyvitamin D:DBP across three samples, deseasonalized and predicted in random intercept model. Maternal mid-pregnancy samples (around week 18 of pregnancy): 385 cases, 519 controls. Maternal postpartum samples (day 0–7): 374 cases, 516 controls. Cord blood: 393 cases, 538 controls. Odds ratios adjusted for maternal celiac disease, sex, age by end of study and celiac disease-associated human leukocyte antigen (HLA) haplotype.
(DOCX)

S1 Table Description of children participating in the Norwegian Mother and Child Cohort Study (MoBa) and children selected for case-control study, including children selected but not included in the case-control study due to lack of available blood samples. * Missing for 11,035 participants.
^**†**^ Missing for 10,900 participants.
^**‡**^ BMI, body mass index (kg/m^2^). Missing information for 13,307 participants.
^***§***^ P-value for trend = 0.69, odds ratio 0.98 (95% CI 0.91 to 1.06) comparing cases vs controls in MoBa cohort. Missing for 37,546 participants.
^**||**^ Missing for 13,090 participants.
^¶^ Odds ratio 1.22 (95% CI, 1.00 to 1.49) for celiac disease for children born March-May compared with December-February in the MoBa cohort.
(DOCX)

S2 Table Odds ratio for celiac disease by vitamin D receptor genotype, and adjusted for vitamin D deficiency genotype score and HLA-type. * rs1544410 (correspondence to BsmI restriction AA = BB, AG = bB, GG = bb).
^***†***^ The rs11568820 SNP is in a sequence containing a binding site for (the transcription factor) CFX2.
^‡^ Odds ratio adjusted for vitamin D genotype score and HLA-type:
5 SNPs for common variants associated with vitamin D levels (rs10741657 for *CYP2R1*, rs703842 for *CYP27B1*, rs6013897 for *CYP24A1*, rs2282679 for *GC* and rs12785878 for *DHCR7*) by number of risk alleles.
(DOCX)

S3 Table Odds ratio for celiac disease with sensitivity analyses, additional adjustments and subgroup analyses. CI = confidence interval
*Main model with logistic regression adjusted for sex, age at end of study, maternal celiac disease, HLA genotype and non-HLA risk genotype score.
^†^ Duration of full breastfeeding, defined as breastmilk without added formula or solid foods, in categories <4, 4–5 and ≥6 months.
^‡^ Pre-pregnant BMI (kg/m^2^) in categories <20, 20–25, 25–30 and >30.
The mother has indicated whether her first tongue is Norwegian or other languages.
^§^ Odds ratio per quartile increase in maternal vitamin A intake and later celiac disease 1.02 (95% CI 0.85 to 1.22, p for trend 0.82).
P = 0.27 for interaction between vitamin D concentration and quartiles of vitamin A intake.
(DOCX)
