# Supplementary material for: Maternal and neonatal vitamin D status, genotype and childhood celiac disease
Source: PLoS One. 2017 Jul 7;12(7):e0179080. doi: 10.1371/journal.pone.0179080 (PMC5501391; doi:10.1371/journal.pone.0179080)
Supplement: S1 Fig — Risk for offspring celiac disease by centiles 25-hydroxyvitamin D modeled for gestational week 25 (A) and for individual sample points (B-D). Density plot for 25-hydroxyvitamin D concentrations among cases (solid line) and controls (dashed line) modeled for gestational week 25 (E) and for individual sample points (F-H). (DOCX) [file pone.0179080.s001.docx]

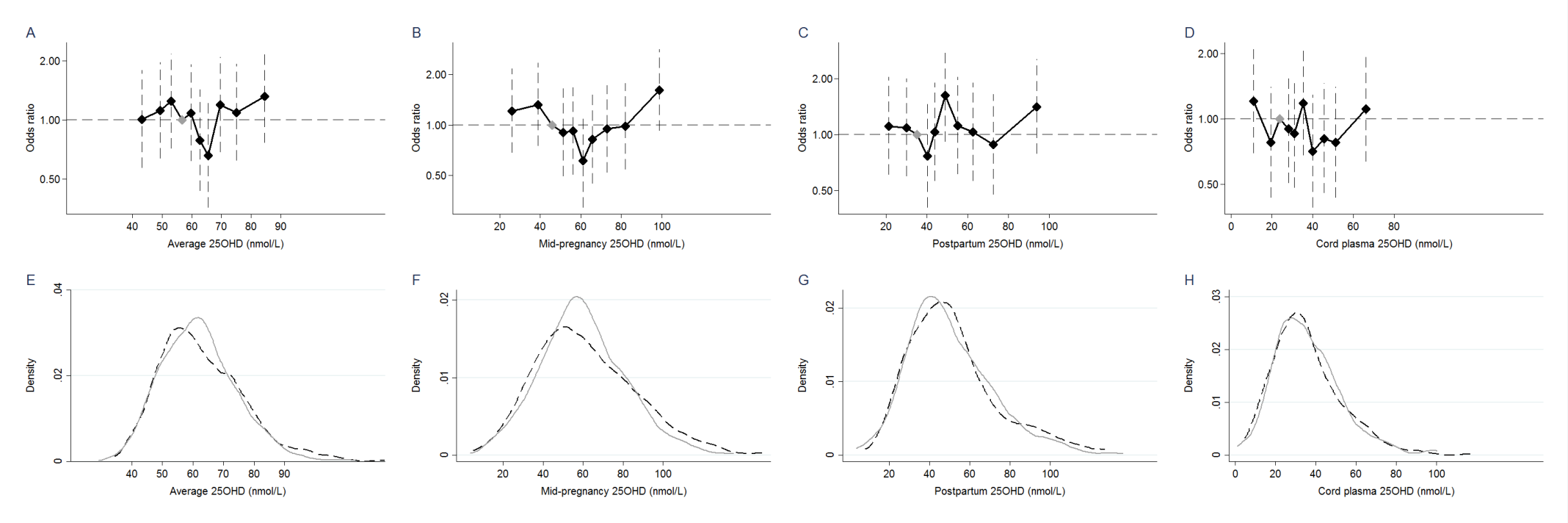


**S1 Fig: Risk for offspring celiac disease by centiles 25-hydroxyvitamin D modeled for gestational week 25 based on all available measurements (A) and for individual sample points (B-D). Density plot for 25-hydroxyvitamin D concentrations among cases (solid line) and controls (dashed line) modeled for gestational week 25 based on all available measurements (E) and for individual sample points (F-H).**
