# Supplementary material for: Maternal and neonatal vitamin D status, genotype and childhood celiac disease
Source: PLoS One. 2017 Jul 7;12(7):e0179080. doi: 10.1371/journal.pone.0179080 (PMC5501391; doi:10.1371/journal.pone.0179080)
Supplement: S2 Fig — DBP, D-vitamin binding protein; aOR, adjusted odds ratio; OR, odds ratio; 95% CI, 95% confidence interval. The primary analysis used the modelled mean ratio of 25-hydroxyvitamin D:DBP across three samples, deseasonalized and predicted in random intercept model. Maternal mid-pregnancy samples (around week 18 of pregnancy): 385 cases, 519 controls. Maternal postpartum samples (day 0–7): 374 cases, 516 controls. Cord blood: 393 cases, 538 controls. Odds ratios adjusted for maternal celiac disease, sex, age by end of study and celiac disease-associated human leukocyte antigen (HLA) haplotype. (DOCX) [file pone.0179080.s002.docx]

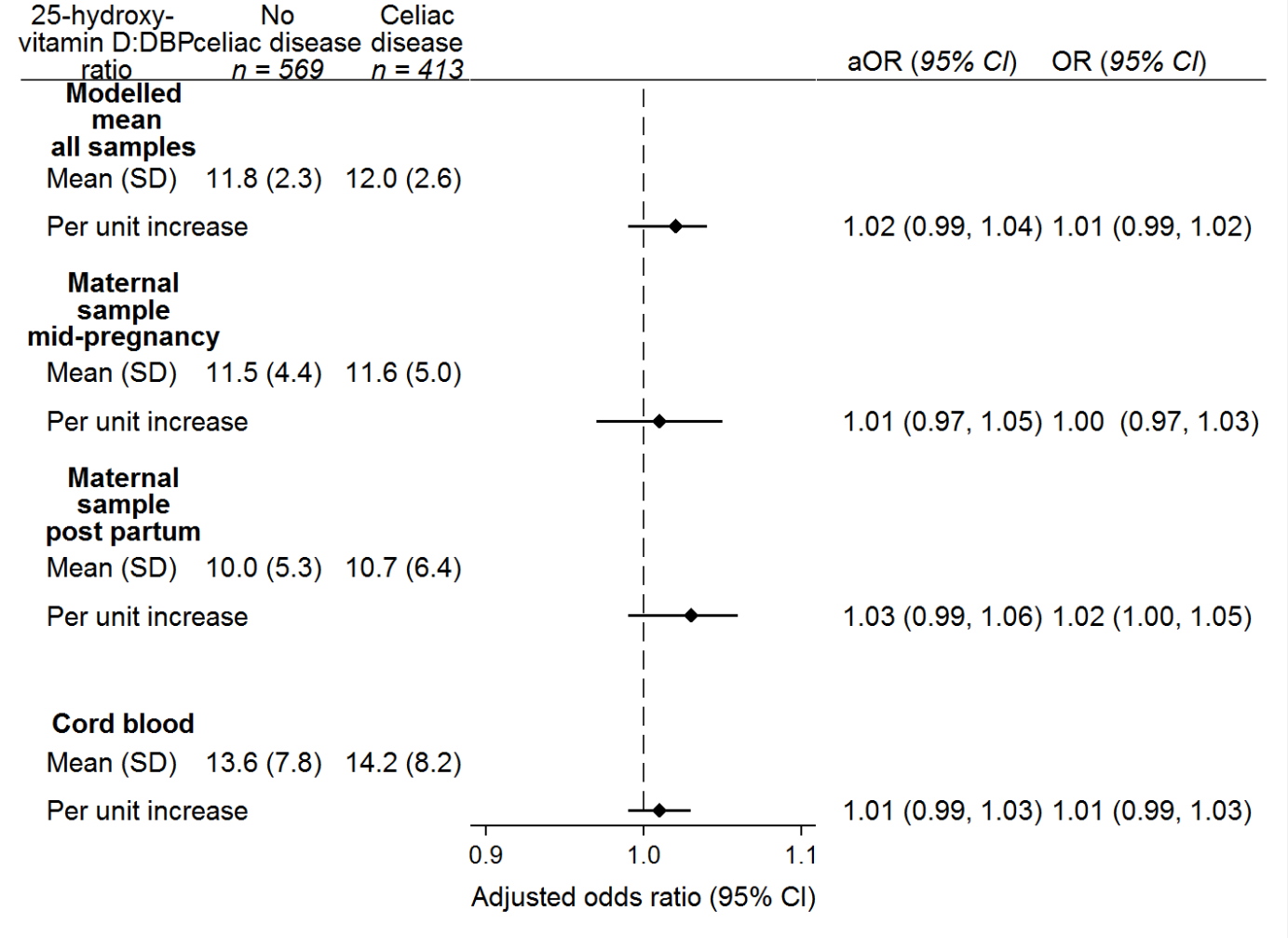


**S2 Fig: Odds ratios for offspring celiac disease according to ratio of**

**25-hydroxyvitamin D:D-vitamin binding protein for week 25 and for individual time points.**

DBP, D-vitamin binding protein; aOR, adjusted odds ratio; OR, odds ratio; 95% CI, 95% confidence interval.

The primary analysis used the modelled mean ratio of 25-hydroxyvitamin D:DBP across three samples, deseasonalized and predicted in random intercept model.

Maternal mid-pregnancy samples (around week 18 of pregnancy): 385 cases, 519 controls.

Maternal postpartum samples (day 0-7): 374 cases, 516 controls.

Cord blood: 393 cases, 538 controls.

Odds ratios adjusted for maternal celiac disease, sex, age by end of study and celiac disease-associated human leukocyte antigen (HLA) haplotype.
