# Supplementary material for: Maternal and neonatal vitamin D status, genotype and childhood celiac disease
Source: PLoS One. 2017 Jul 7;12(7):e0179080. doi: 10.1371/journal.pone.0179080 (PMC5501391; doi:10.1371/journal.pone.0179080)
Supplement: S1 Table — * Missing for 11,035 participants. † Missing for 10,900 participants. ‡ BMI, body mass index (kg/m2). Missing information for 13,307 participants. § P-value for trend = 0.69, odds ratio 0.98 (95% CI 0.91 to 1.06) comparing cases vs controls in MoBa cohort. Missing for 37,546 participants. || Missing for 13,090 participants. ¶ Odds ratio 1.22 (95% CI, 1.00 to 1.49) for celiac disease for children born March-May compared with December-February in the MoBa cohort. (DOCX) [file pone.0179080.s003.docx]

**S1 Table. Description of children participating in the Norwegian Mother and Child Cohort Study (MoBa) and children selected for case-control study, including children selected but not included in the case-control study due to lack of available blood samples.**

|  | MoBa cohort | | Selected for case-control study | | | |
| --- | --- | --- | --- | --- | --- | --- |
|  | Celiac disease | | Celiac disease | | | |
|  | No (112,084) | Yes  (784) | No  (n=996) | | Yes  (n=784) | |
|  |  |  | Included  (n=570) | Not included (n=426) | Included  (n=416) | Not included (n=368) |
| Mother (n, %) |  |  |  |  |  |  |
| *Maternal age* |  |  |  |  |  |  |
| <25 | 13,037 (12) | 89 (11) | 71 (12) | 52 (12) | 50 (12) | 39 (11) |
| 25-34 | 79,378 (71) | 575 (73) | 401 (70) | 286 (67) | 312 (75) | 259 (71) |
| ≥35 | 19,669 (18) | 120 (15) | 98 (17) | 88 (21) | 54 (13) | 66 (18) |
| *Parity* |  |  |  |  |  |  |
| 0 [first child] | 49,395 (44) | 330 (42) | 247 (43) | 190 (45) | 169 (41) | 159 (44) |
| 1 | 40,234 (36) | 311 (40) | 212 (37) | 147 (35) | 170 (41) | 140 (39) |
| ≥2 | 22,455 (20) | 143 (18) | 111 (20) | 89 (21) | 77 (19) | 65 (18) |
| *Cesarean delivery* |  |  |  |  |  |  |
| Yes | 16,998 (15) | 101 (13) | 59 (10) | 94 (22) | 48 (12) | 53 (15) |
| *Maternal education^*^* |  |  |  |  |  |  |
| ≤12 years | 38,002 (38) | 253 (35) | 204 (39) | 144 (40) | 146 (37) | 105 (33) |
| 13-15 years | 40,618 (40) | 290 (40) | 213 (40) | 126 (35) | 168 (42) | 121 (38) |
| ≥15 years | 22,494 (22) | 176 (25) | 111 (21) | 89 (25) | 84 (21) | 91 (29) |
| *Smoking in pregnancy^†^* |  |  |  |  |  |  |
| No | 91,355 (90) | 671 (93) | 465 (88) | 320 (90) | 371 (92) | 296 (93) |
| Occasionally | 1,932 (2) | 12 (2) | 15 (3) | 5 (1) | 8 (2) | 4 (1) |
| Daily | 7,957 (8) | 41 (6) | 51 (10) | 32 (9) | 23 (6) | 18 (6) |
| *Pre-pregnancy BMI^‡^* |  |  |  |  |  |  |
| <20 | 12,563 (13) | 94 (13) | 58 (11) | 55 (16) | 51 (13) | 42 (14) |
| 20-24.9 | 55,215 (56) | 400 (57) | 313 (61) | 181 (51) | 217 (55) | 182 (59) |
| 25-29.9 | 21,611 (22) | 145 (21) | 108 (21) | 80 (23) | 83 (21) | 61 (20) |
| ≥30 | 9,467 (10) | 66 (9) | 38 (7) | 36 (10) | 41 (11) | 25 (8) |
| *Maternal celiac disease* |  |  |  |  |  |  |
|  | 796 (0.7) | 72 (9) | 2 (0.4) | 4 (1) | 39 (9) | 33 (9) |
| *Vitamin D intake (μg/d) in pregnancy, quartiles^§^* |  |  |  |  |  |  |
| <5.1 | 18,677 (25) | 141 (25) | 99 (26) | 74 (27) | 89 (29) | 50 (20) |
| 5.1-8.2 | 18,694 (25) | 142 (25) | 92 (24) | 68 (25) | 65 (22) | 77 (30) |
| 8.2-13.2 | 18,697 (25) | 146 (26) | 102 (26) | 63 (23) | 75 (25) | 70 (28) |
| >13.2 | 18,693 (25) | 132 (24) | 94 (24) | 67 (25) | 74 (24) | 57 (22) |
| *Norwegian origin*^\|\|^ |  |  |  |  |  |  |
|  | 92,993 (94) | 680 (97) | 491 (94) | 334 (94) | 376 (97) | 300 (97) |
|  | **MoBa cohort** | | **Selected for case-control study** | | | |
|  | Celiac disease | | Celiac disease | | | |
|  | No (112,084) | Yes  (784) | No  (n=996) | | Yes  (n=784) | |
|  |  |  | Included  (n=570) | Not included (n=426) | Included  (n=416) | Not included (n=368) |
| Child |  |  |  |  |  |  |
| *Female, (%)* |  |  |  |  |  |  |
|  | 54,545 (49) | 480 (61) | 280 (49) | 222 (52) | 250 (60) | 226 (62) |
| *Age at end of follow-up (mean/SD)* |  |  |  |  |  |  |
|  | 8.4 (2.2) | 8.7 (2.1) | 8.7 (2.3) | 8.0 (2.1) | 9.0 (2.0) | 8.4 (2.1) |
| *Birthweight, kg* |  |  |  |  |  |  |
| <2.5 | 4,700 (4) | 24 (3) | 8 (1) | 35 (8) | 8 (2) | 16 (4) |
| 2.5-3.49 | 42,700 (38) | 340 (43) | 238 (42) | 161 (38) | 176 (42) | 163 (45) |
| 3.5-4.5 | 59,724 (53) | 389 (50) | 295 (52) | 217 (51) | 215 (52) | 171 (47) |
| >4.5 | 4,898 (4) | 31 (4) | 29 (5) | 13 (3) | 17 (4) | 14 (4) |
| *Prematurity (<37 weeks)* |  |  |  |  |  |  |
|  | 6,961 (6) | 38 (5) | 19 (3) | 43 (10) | 16 (4) | 22 (6) |
| *Season of birth^¶^* |  |  |  |  |  |  |
| Dec.-February | 26,176 (23) | 166 (21) | 142 (25) | 95 (22) | 90 (22) | 76 (21) |
| March-May | 30,003 (27) | 232 (30) | 154 (27) | 114 (27) | 115 (28) | 114 (31) |
| June-August | 29,477 (26) | 200 (26) | 138 (24) | 109 (26) | 106 (26) | 93 (26) |
| Sep.-November | 26,428 (24) | 186 (24) | 136 (24) | 107 (25) | 105 (25) | 81 (22) |

^*^ Missing for 11,035 participants.

**^†^** Missing for 10,900 participants.

**^‡^** BMI, body mass index (kg/m^2^). Missing information for 13,307 participants.

***^§^*** P-value for trend = 0.69, odds ratio 0.98 (95% CI 0.91 to 1.06) comparing cases vs controls in MoBa cohort. Missing for 37,546 participants.

**^||^** Missing for 13,090 participants.

^¶^ Odds ratio 1.22 (95% CI, 1.00 to 1.49) for celiac disease for children born March-May compared with December-February in the MoBa cohort.
