# Supplementary material for: Maternal and neonatal vitamin D status, genotype and childhood celiac disease
Source: PLoS One. 2017 Jul 7;12(7):e0179080. doi: 10.1371/journal.pone.0179080 (PMC5501391; doi:10.1371/journal.pone.0179080)
Supplement: S2 Table — * rs1544410 (correspondence to BsmI restriction AA = BB, AG = bB, GG = bb). † The rs11568820 SNP is in a sequence containing a binding site for (the transcription factor) CFX2. ‡ Odds ratio adjusted for vitamin D genotype score and HLA-type: 5 SNPs for common variants associated with vitamin D levels (rs10741657 for CYP2R1, rs703842 for CYP27B1, rs6013897 for CYP24A1, rs2282679 for GC and rs12785878 for DHCR7) by number of risk alleles. (DOCX) [file pone.0179080.s004.docx]

**S2 Table: Odds ratio for celiac disease by vitamin D receptor genotype, and adjusted for vitamin D deficiency genotype score and HLA-type.**

|  | Celiac disease | | Crude odds ratio for celiac disease  (95% CI) | Adjusted^‡^ odds ratio  (95% CI) |
| --- | --- | --- | --- | --- |
| *VDR genotype* | **No** | **Yes** |  |  |
| *rs1544410^*^* | n=547 (%) | n=394 (%) |  |  |
| AA  AG  GG | 101 (18.5)  254 (46.3)  193 (35.3) | 74 (18.8)  193 (49.0)   1. (2.2) | 1.00 (ref.)  1.04 (0.73, 1.48)  0.90 (0.62, 1.31) | 1.00 (ref.)  1.11 (0.72, 1.70)  0.83 (0.53, 1.29) |
| *rs11568820 ^†^* | n=548 (%) | n=394 (%) |  |  |
| AA  AG  GG | 22 (4.0)  135 (24.6)  391 (71.4) | 13 (3.3)  126 (32.0)  255 (64.7) | 1.00 (ref.)  1.58 (0.76, 3.27)  1.10 (0.55, 2.23) | 1.00 (ref.)  1.40 (0.59, 3.33)  1.10 (0.48, 2.55) |

***^*^*** rs1544410 (correspondence to BsmI restriction AA=BB, AG=bB, GG=bb).

***^†^*** The rs11568820 SNP is in a sequence containing a binding site for (the transcription factor) CFX2.

^‡^ Odds ratio adjusted for vitamin D genotype score and HLA-type:

5 SNPs for common variants associated with vitamin D levels (rs10741657 for *CYP2R1,* rs703842 for *CYP27B1*, rs6013897 for *CYP24A1,* rs2282679 for *GC* and rs12785878 for *DHCR7*) by number of risk alleles.
