# Supplementary material for: Maternal and neonatal vitamin D status, genotype and childhood celiac disease
Source: PLoS One. 2017 Jul 7;12(7):e0179080. doi: 10.1371/journal.pone.0179080 (PMC5501391; doi:10.1371/journal.pone.0179080)
Supplement: S3 Table — CI = confidence interval *Main model with logistic regression adjusted for sex, age at end of study, maternal celiac disease, HLA genotype and non-HLA risk genotype score. † Duration of full breastfeeding, defined as breastmilk without added formula or solid foods, in categories <4, 4–5 and ≥6 months. ‡ Pre-pregnant BMI (kg/m2) in categories <20, 20–25, 25–30 and >30. The mother has indicated whether her first tongue is Norwegian or other languages. § Odds ratio per quartile increase in maternal vitamin A intake and later celiac disease 1.02 (95% CI 0.85 to 1.22, p for trend 0.82). P = 0.27 for interaction between vitamin D concentration and quartiles of vitamin A intake. (DOCX) [file pone.0179080.s005.docx]

**S3 Table: Odds ratio for celiac disease with sensitivity analyses, additional adjustments and subgroup analyses.**

|  | Celiac disease | | Adjusted odds ratio  (95% CI) |
| --- | --- | --- | --- |
|  | No  (n=570) | Yes (n=416) |  |
| Main model^*^ | 570 | 416 | 1.06 (0.93, 1.20) |
| *Sensitivity analysis* |  |  |  |
| Main model without adjustment for season | 570 | 416 | 1.05 (0.91, 1.22) |
| Parental-confirmed cases only | 570 | 262 | 1.12 (0.97, 1.30) |
| *Additional adjustment* |  |  |  |
| Duration of full breastfeeding^†^ | 469 | 363 | 1.02 (0.89, 1.17) |
| Pre-pregnant BMI^‡^ | 517 | 392 | 1.06 (0.93, 1.21) |
| Maternal origin | 521 | 389 | 1.04 (0.91, 1.18) |
| Maternal vitamin A intake^§^ | 387 | 303 | 0.96 (0.83, 1.12) |
| *Subgroup analyses* |  |  |  |
| Females | 280 | 250 | 1.00 (0.85, 1.19) |
| Males | 290 | 166 | 1.13 (0.94, 1.37) |
| Age of diagnosis <4 years |  | 114 | 1.13 (0.93, 1.37) |
| Age of diagnosis ≥4 years |  | 146 | 1.14 (0.95, 1.36) |
| HLA high risk group | 19 | 116 | 1.34 (0.84, 2.04) |
| HLA moderate risk group | 284 | 268 | 1.06 (0.92, 1.21) |

CI=confidence interval

^*^Main model with logistic regression adjusted for sex, age at end of study, maternal celiac disease, HLA genotype and non-HLA risk genotype score.

^†^ Duration of full breastfeeding, defined as breastmilk without added formula or solid foods, in categories <4, 4-5 and ≥6 months.

^‡^ Pre-pregnant BMI (kg/m^2^) in categories <20, 20-25, 25-30 and >30.

The mother has indicated whether her first tongue is Norwegian or other languages.

^§^ Odds ratio per quartile increase in maternal vitamin A intake and later celiac disease 1.02 (95% CI 0.85 to 1.22, p for trend 0.82).

P=0.27 for interaction between vitamin D concentration and quartiles of vitamin A intake.
